# Three-dimensional visualizations from a dataset of immunohistochemical stained serial sections of human brain tissue containing tuberculosis related granulomas

**DOI:** 10.1016/j.dib.2020.106532

**Published:** 2020-11-14

**Authors:** Stefan-Dan Zaharie, Daniel J. Franken, Martijn van der Kuip, Sabine van Elsland, Bernadette S. de Bakker, Jaco Hagoort, Sanna L. Roest, Carmen S. van Dam, Carlie Timmers, Regan Solomons, Ronald van Toorn, Mariana Kruger, A. Marceline van Furth

**Affiliations:** 1 Department of Anatomical Pathology, Faculty of Medicine and Health Sciences, Stellenbosch University and National Health Laboratory Services, Francie Van Zijl Dr, Parrow, Tygerberg Hospital, Cape Town 7505, South Africa; 2 Department of Paediatric Infectious Diseases and Immunology, Amsterdam Infection & Immunity Institute, Amsterdam UMC, Vrije Universiteit Amsterdam, De Boelelaan 1117, 1081 HV Amsterdam, the Netherlands; 3 Department of Paediatrics and Child Health, Faculty of Medicine and Health Sciences, Stellenbosch University, Francie van Zijl Dr, Parrow, Tygerberg Hospital, Cape Town 7505, South Africa; 4 Department of Medical Biology, Section Clinical Anatomy & Embryology, Amsterdam University Medical Centers, University of Amsterdam, Meibergdreef 9, 1105 AZ Amsterdam Zuidoost, the Netherlands

**Keywords:** Central nervous system tuberculosis, Tuberculous meningitis, Granuloma, Rich-focus, 3D-reconstruction

## Abstract

This data article presents datasets associated with the research article entitled “The immunological architecture of granulomatous inflammation in central nervous system tuberculosis’’ (Zaharie et al., 2020). The morphology of tuberculosis related granulomas within the central nervous system of human patients was visualized in six different three-dimensional (3D) models. Post-mortem, formalin fixed and paraffin embedded specimens from deceased tuberculous meningitis patients were immunohistochemically stained and 800 serial histologically stained sections were acquired. Images from all sections were obtained with an Olympus BX43 light microscope and structures were identified, labeled and made three-dimensional. The interactive 3D-models allows the user to directly visualize the morphology of the granulomas and to understand the localization of the granulomas. The 3D-models can be used for multiple purposes and provide both an educational source as a gold standard for further animal studies, human research and the development of in silico models on the topic of central nervous system tuberculosis.

## Specifications Table

**Table:** 

Subject	Health and medical sciences.
Specific subject area	Infectious diseases.
Type of data	Interactive 3D-portable document format (3D-PDF) files. These files contain 3D models, example images of histological sections, clinical patient data and a usage guide.
How data were acquired	Three-dimensional models of granulomas were made using histological images derived from post-mortem human central nervous system tissue, which were acquired with the Olympus BX43 light microscope equipped with Olympus soft imaging solutions GMBH camera model SC100.
Data format	Analyzed Portable document format (PDF) files containing 3D models. 3D interaction is only possible with Adobe Acrobat Reader or Adobe Acrobat Pro on MS Windows or Mac Os.
Parameters for data collection	Well-preserved human post-mortem specimens from the central nervous system of tuberculous meningitis (TBM) patients were fixed with formalin and embedded in paraffin and were selected for imaging.
Description of data collection	Three-dimensional models of tuberculous granulomas were visualized from histological images.
Data source location	Stellenbosch University, Cape Town, South Africa.
Data accessibility	Data is provided as Supplementary materials directly within this article.
Related research article	S.D. Zaharie, D.J. Franken, M. van der Kuip, S. van Elsland, B.S. de Bakker, J. Hagoort, S.L. Roest, C.S. van Dam, C. Timmers, R. Solomons, R. van Toorn, M. Kruger, A.M. van Furth, The immunological architecture of granulomatous inflammation in central nervous system tuberculosis, Tuberculosis 2020;125:102016. https://doi.org/10.1016/j.tube.2020.102016

## Value of the Data

• The presented datasets provide three-dimensional (3D) models of human brain tissue containing tuberculous related granuloma.
• The 3D datasets can be used for multiple purposes including interactive viewing of the granulomas within the central nervous system, the localization of the primary granulomas and observation of the morphology of granulomas. Especially, neovascularization within and surrounding the granulomas can well be observed within the 3D models which can be used by researchers working on mycobacterial brain entry.
• The 3D dataset and immunohistochemistry data of tuberculous granulomas in the human central nervous system can serve as a gold standard for further studies on granulomas within animal models (e.g. zebrafish models, macaques) or the development of agent-based (in silico) models.
• The 3D models can be used for educational purposes to provide insight in the morphology of central nervous system granulomas.

## 1 Data Description

The presented 3D-portable document format (3D-PDF) files illustrate the spatial relationship and morphology of three different types of tuberculosis related granulomas within the central nervous system of human patients [1]. Each 3D-PDF consists of three or four pages; the information page, the 3D model, the immunohistochemical staining, the clinical data. A user interface was designed with a structure tree providing options to select each separate structure and to ‘show’, ‘transparent’ and ‘hide’ the selected structure. Some predefined views were created as examples to direct the attention of the user to certain structures. The PDF files should be viewed in Adobe Reader® available from http://www.adobe.com/downloads/. 3D interaction is only possible on MS Windows or Mac OS. JavaScript must be enabled. The morphology as well as analysis and some images shown in the supplementary files are also presented within the original manuscript as still images [1].

## 2 Experimental Design, Materials and Methods

### 2.1 Patient cohort

This human neuropathological retrospective study involved a unique cohort of infants, children, and adults, who were diagnosed with TBM between 1975 and 2012 at Tygerberg Hospital, Cape Town, South Africa. We examined 439 post-mortem and 24 biopsy-derived brain specimens obtained from 84 patients. The specimens were formalin-fixed and paraffin-embedded (FFPE) (mean: 5; range 1–16 blocks per patient). The patient's demographic information and clinical data were retrieved from medical files. All patients received treatment according to the guidelines at the time of hospitalization.

### 2.2 Details of the immunohistochemistry

To ensure a constant and high quality, immunohistochemical staining was performed using the automated Leica BOND-III machine and appropriate controls were used to validate and optimize the stains. After dewaxing using bond dewax solution (Leica, AR9222) at 72 °C, tissue sections were washed with bond wash solution (Leica, AR9590) and treated for 20 min with epitope retrieval (ER) solutions (Leica ER1, AR9961; Leica ER2, AR9640). Sections were incubated with a primary antibody for 15 min at room temperature. Counterstaining was performed using hematoxylin. Innate immunity was assessed using CD68 for macrophages/microglia (1:1000 Dako, M0814), myeloperoxidase (MPO) for neutrophils (1:2000 Dako, A0398), and CD11b for microglia/neutrophils (1:100 Abcam, EP1345Y). Adaptive humoral immunity was assessed using CD20 for B-cells (1:500 Dako, M0755). Interferon (IFN)-γ (1:100 Abcam, ab9657) and tumor necrosis factor (TNF)-α (1:200 Abcam, ab6671) immunohistochemical stains were performed. More immunohistochemical staining are provided elsewhere [1]. Bound antibodies were detected with a polymer-based detection kit and 3,3’diaminobenzidine (DAB)-chromogen (Leica Bond Polymer Refine: DS9800).

### 2.3 Three-dimensional modeling

3D-models were created to facilitate understanding of the topographic relation of granulomas with meninges, brain parenchyma and blood vessels. The degree of detail required for 3D-reconstruction of distinct structures of intracranial granulomas is currently impossible to obtain with non-invasive techniques. Therefore, we randomly selected four FFPE brain specimens, containing one or two complete and well-circumscribed granulomas, to generate 3D-reconstructions of six different granulomas as described [1]. FFPE brain specimens were serially sectioned by a Leica SM2010R Slicing microtome at 4 μm and stained with hematoxylin-eosin and reticulin silver. The methods to generate 3D-reconstructions are previously described by de Bakker et al. [2]. The histological slides were analyzed using an Olympus BX43 light microscope and photos were taken using the Olympus soft imaging solutions GMBH camera model SC100. Digital images were acquired from 800 serial histologically stained sections with a resolution of 3840 × 2748 pixels. After image acquisition, all images were converted with IrfanView (http://www.irfanview.com) into greyscale ‘TIFF’ format and imported into an Amira® software package (Amira 5.6, Thermo Fischer Scientific, http://www.amira.com).

The reconstruction procedure in Amira is as follows: the greyscale images were automatically aligned by using the Align Slices module with the least-square mode selected. The automatic alignment was manually adjusted when needed. For the 3D reconstructions we used 6 stacks of aligned serial images ranging from 43 to 231 images. These stacks were saved as Amira Binary (.am) file. Segmentation was performed by using the Segmentation Editor. Drawing was made easier by using a Bamboo tablet and pen (http://www.wacom.com). Structures of interest, as brain parenchyma, meninges, blood vessels, granulomas, necrotized areas and giant cells were manually segmented while the high-resolution full-color dataset was displayed on a second computer screen. The segmentation results were saved as Amira label (.am) files. From these label files, 3D surface models were created with the GenerateSurface module. When surfaces showed irregularities, due to section deformation, the segmentations were manually corrected to get a more smooth and morphological correct structure shape. The surfaces were simplified with the Simplification editor. The number of resulting triangles was chosen by the type of structure, to be sure that all desired details were still visible but with a minimal number of triangles. As a final step, the surfaces were smoothed with the SmoothSurface module. The surfaces were saved as Surface Binary (.surf) file.

Interactive 3D-portable document format (3D-PDF) files containing all modeled structures were created as previously described by Warmbrunn et al. [3] and include the following steps: Amira surface (.surf) files were converted to .u3d files with Fiji (http://imagej.net/fiji). In Deep Exploration (version 6.5 CSE, part of Corel DESIGNER Technical Suite ×5 http://www.corel.com) small corrections to surfaces, colors, names and grouping of structures were made and again saved as .u3d file.

The final layout containing all non-3D content was created with MS PowerPoint (http://www.microsoft.com) and saved as a 3-page (.pdf) file per reconstruction. In Adobe Acrobat XI Pro (http://www.adobe.com) the .u3d files were imported and placed as a 3D object on page 2 of each pdf file. The pages are made visible as tabs. By using custom made Acrobat JavaScripts, a user interface was created for easier viewing and selecting of structures and tabs.

3D interaction of these 3D-pdf files is only possible with a recent version of Adobe Acrobat Reader or Pro (X or higher, http://www.adobe.com) on MS Windows or MacOS systems, with JavaScript and playing of 3D content enabled. Using the interactive 3D-PDF files, we were able to obtain a good understanding of the spatial relations between the granuloma and their surrounding structures, such as the meninges and blood vessels.

## Ethics Statement

The study was approved by the Health Research Ethics Committee of the Faculty of Health and Medical Sciences, Stellenbosch University, Cape Town, Western Cape, South Africa (ethics approval numbers S12/11/298 and N09/07/185). The post-mortem material was collected after a full autopsy was performed immediately after the patient's demise. In South Africa, consent from the relatives for a post-mortem autopsy has always been mandatory including the period of 1975 to 2012 when the material for the present study was collected. Informed consent for this present study was waived by the Stellenbosch University Human Research Ethics Committee. The waiver of consent has been granted due to the difficulty in tracing the families of the deceased patients, as well as due to the scientific importance of the further mentioned brain tissue material. Data analyses were done anonymously, using unique study numbers.

## Author contributions

All authors had full access to all the data in the study and take responsibility for the integrity of the data and the accuracy of the data analysis. DJF, BSdB, JH, AMvF, MvdK and SDZ designed the project and wrote the manuscript. DJF, BSdB and JH were responsible for the 3D reconstructions and modelling. SvE was the project coordinator and responsible for the data management. SDZ, DJF, SLR, CSvD and CT analyzed the (immuno)histology data. MK, RvT and RS were involved in the assessment of clinical data.

## Declaration of Competing Interest

The authors declare that they have no known competing financial interests or personal relationships which have, or could be perceived to have, influenced the work reported in this article.

## Appendix Supplementary materials

Image, application 1
