# Supplementary material for: Three-dimensional visualizations from a dataset of immunohistochemical stained serial sections of human brain tissue containing tuberculosis related granulomas
Source: Data Brief. 2020 Nov 14;33:106532. doi: 10.1016/j.dib.2020.106532 (PMC7701168; doi:10.1016/j.dib.2020.106532)
Supplement: Supplementary file 1 [file mmc1.zip › Abscess type granuloma_1.pdf]

Information on the use of this interactive 3D-PDF

Help | 3D model | Immunohistochemistry | Clinical data patient

- Granuloma 1: Rich focus
- Granuloma 2
- Necrosis
- Giant cells
- Blood vessels
- Brain parenchymal surface
- Meninges

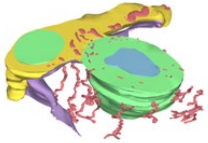
Overview

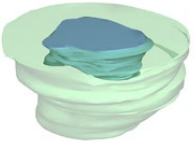
Granuloma

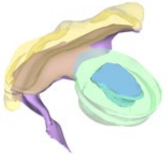
Rich focus

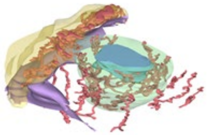
Blood vessels

Amsterdam UMC
University Medical Centers

Selection of structures

The top left panel contains buttons to show or hide structures, or to make them transparent.

button example:

show transparent hide

Interaction with the 3D model

Rotate: Hold left mouse-button and move mouse.
Zoom: Hold right mouse-button and move mouse up or down or scroll.
Translate: Hold left and right mouse-buttons and move mouse.

Selection of preset views

Click on a view button to display the preset view as shown on the button.

Full screen mode

Enter full screen mode: Ctrl + L
Exit full screen mode: Esc

Immunohistochemistry and clinical data patient

Click on the tab “Immunohistochemistry” to display the immunohistochemical stainings of the granuloma.
Click on the tab “Clinical data patient” to display the data of the patient.

Technical notes

This PDF file should be viewed in Adobe Acrobat Reader X or higher. 3D interaction is only possible on MS Windows or Mac OS. Javascript and playing of 3D content must be enabled.
Open Edit, Preferences to ensure the following:

1) In JavaScript: enable Enable Acrobat JavaScript
2) In 3D & Multimedia: enable Enable playing of 3D content
3) In 3D & Multimedia, 3D Tool Options: disable Show 3D Orientation Axis
4) In 3D & Multimedia, Auto-Degrade Options, Optimization Scheme for Low Framerate: select None

Granuloma 1: Abscess granuloma (Rich focus)

Help | 3D model | Immunohistochemistry | Clinical data patient

- Granuloma 1: Rich focus
- Granuloma 2
- Necrosis
- Giant cells
- Blood vessels
- Brain parenchymal surface
- Meninges

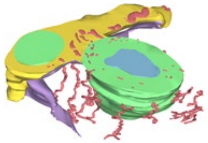

Overview

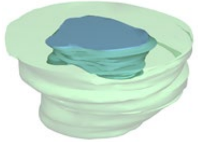

Granuloma

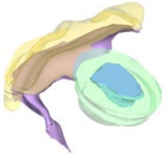

Rich focus

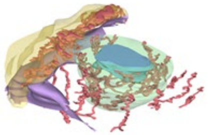

Blood vessels

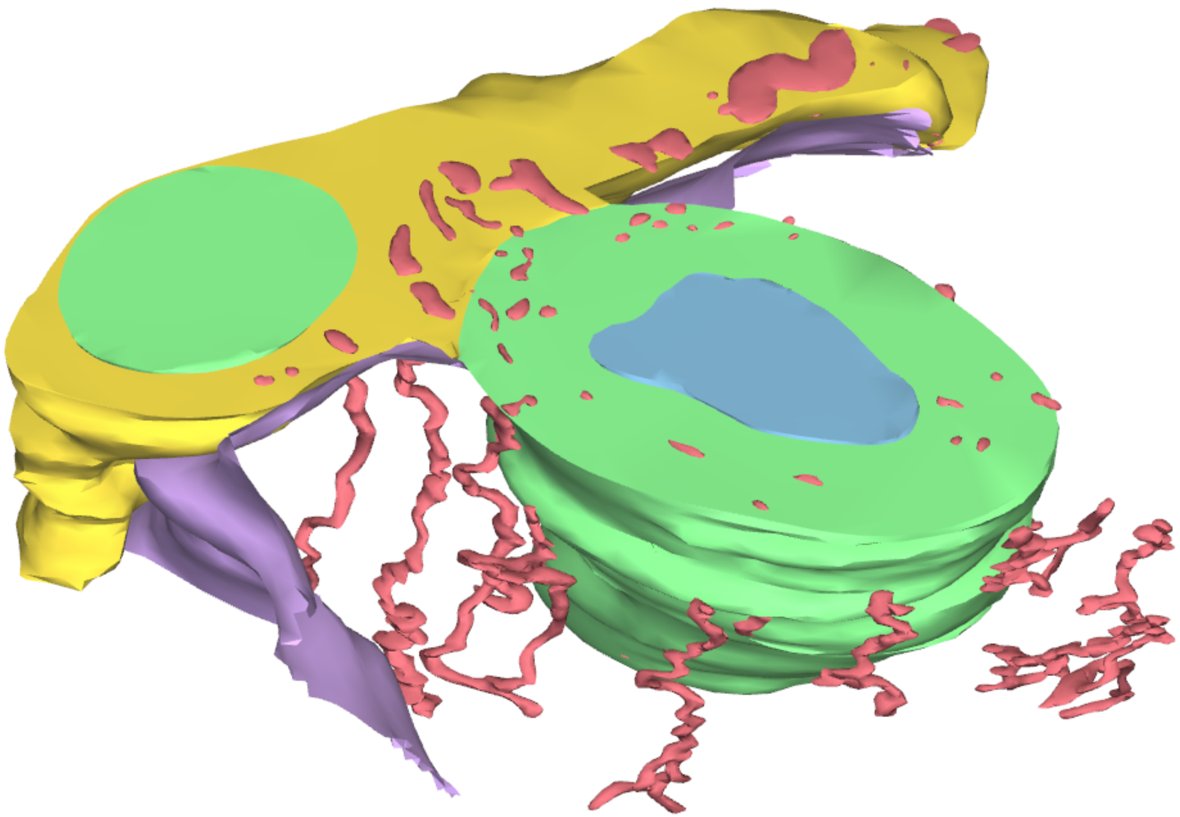

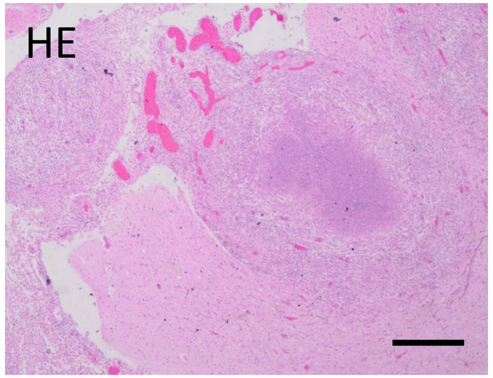

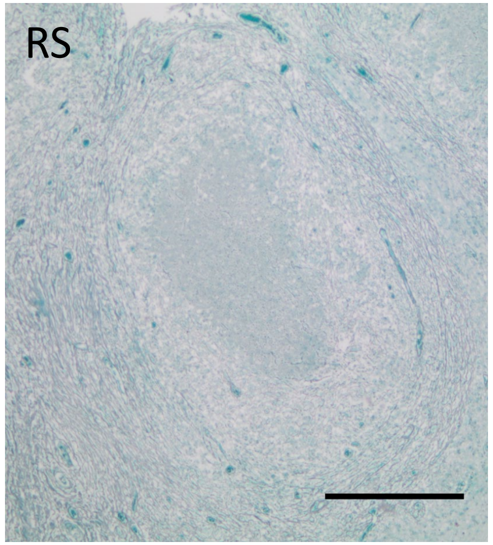

Scale bar = 200 µm

Granuloma 1: immunohistochemistry of abscess granuloma

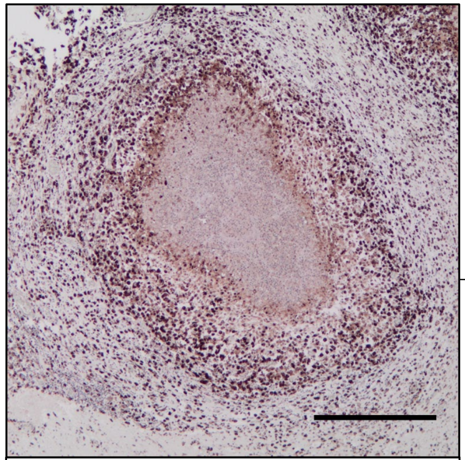

CD68 stains macrophages, microglia, giant cells within cell layers of the granuloma

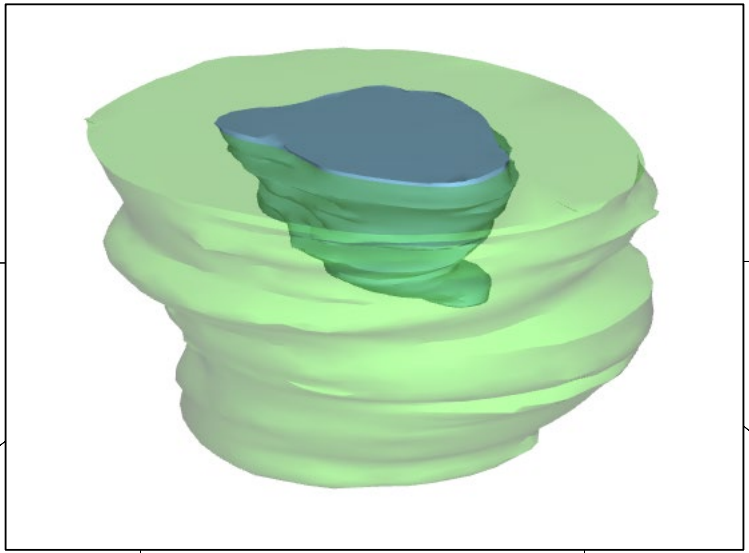

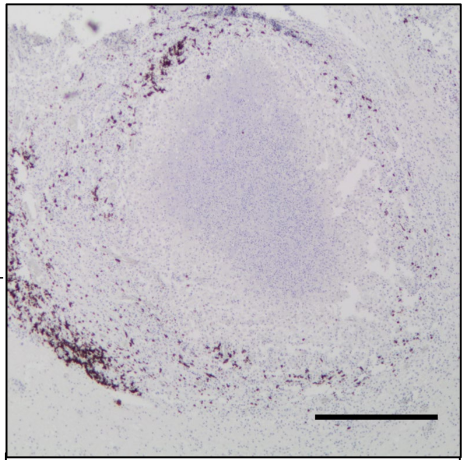

CD20 B-lymphocytes: stains the B-cells in the outer layers of granuloma

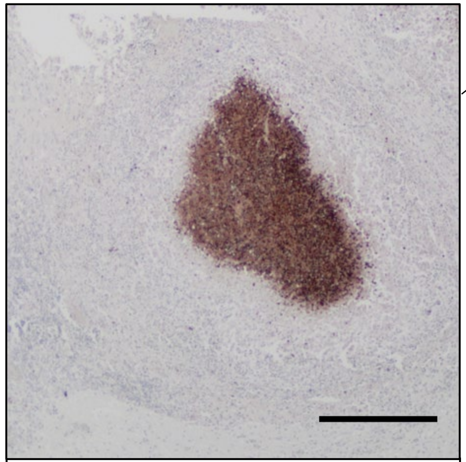

MPO: stains neutrophils within the necrosis

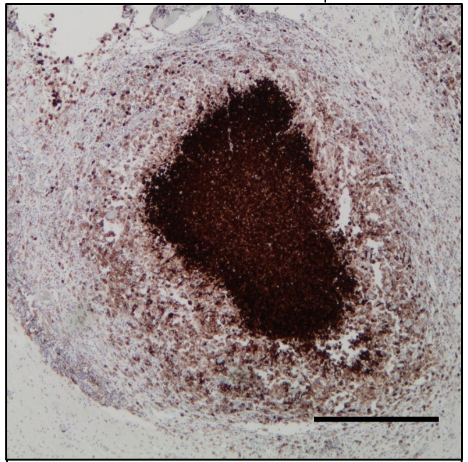

CD11b: stains neutrophils, macrophages, microglia and giant cells within granuloma

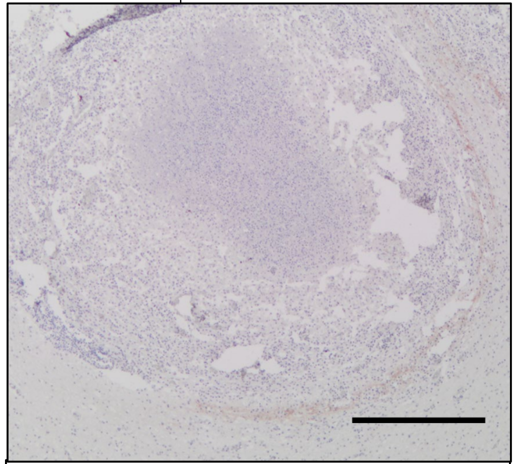

TNF-α: stains tumor necrosis factor alpha surrounding the granuloma

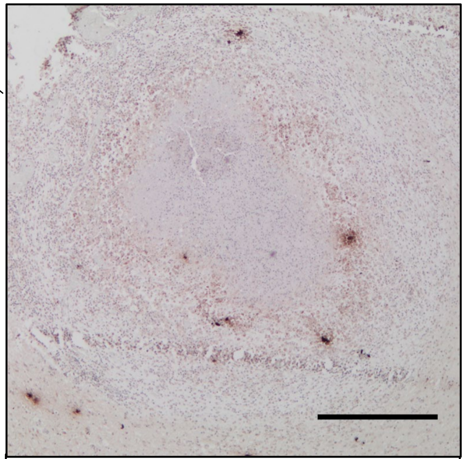

IFN-γ: stains interferon gamma within granuloma with highest concentration surrounding the necrosis

Scale bare = 200 μm

Clinical data of patient (case number 11)

Background	
Year of admission at hospital	1984
Age (month)	18 month
Sex	Female
Clinical information	
Initial presentation	convulsions, weight loss, weakness in both legs, cough for 4 days, drowsiness for 2 days
Physical examination	Stuporose, unreactive left pupil, muscle tone and reflexes normal
Household contacts with TB	Mother and sister of the patient were being treated for tuberculosis
Diagnosis	Probable
TBM Stage[a]	Stage III
Lumbar puncture	lymphocytes 208, protein 3,0g/L , globulin +++, glucose <1 mmol/L
Cerebral imaging	CT (day 4 post treatment): hydrocephalus
Treatment	
Tuberculostatics	Rifampicin, Streptomycin, Isoniazide, Pyrazinamide, Ethionamide, Ethambutol, Penicilline G
Other medication	Prednison, Mucospect, Fenobarbital, Amoxicilline, Domperidon, Bactrim, Acetazolamide, Decadron
Duration	84 days
Additional treatment	A ventriculoperitoneal-shunt was inserted for drainage of the hydrocephalus
Outcome	Death
Post mortem	
Central Nervous system	Meninges at the base of the brain were thickened and had a nodular appearance of tuberculomas. Sections of the brain confirmed severe ventricular dilatation from the hydrocephalus. Caseating subarachnoid tuberculomas were present at the pons of the brain.
Other tissue	Within the lung of the patient a subpleural focus of tuberculosis was present at the oblique fissure of the right lung with purulent effusion within the pleural cavity.

a. Tuberculous meningitis stage is based on the ‘refined’ British Medical Research Council scale (van Toorn 2012)
