# Supplementary material for: Three-dimensional visualizations from a dataset of immunohistochemical stained serial sections of human brain tissue containing tuberculosis related granulomas
Source: Data Brief. 2020 Nov 14;33:106532. doi: 10.1016/j.dib.2020.106532 (PMC7701168; doi:10.1016/j.dib.2020.106532)
Supplement: Supplementary file 1 [file mmc1.zip › Abscess type granuloma_2.pdf]

Information on the use of this interactive 3D-PDF

Granuloma
Necrosis
Giant cells
Blood vessels
Cerebellum
Meninges

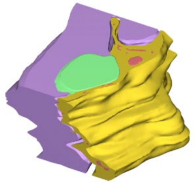

Overview

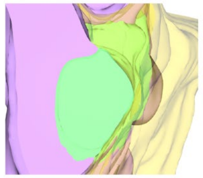

Rich focus

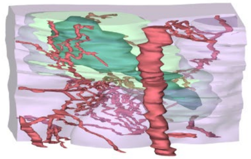

Blood vessels

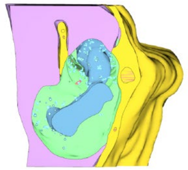

Granuloma layers

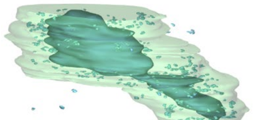

Granuloma

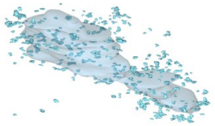

Giant cells

Amsterdam UMC
University Medical Centers

Selection of structures

The top left panel contains buttons to show or hide structures, or to make them transparent.

button example:
show transparent hide

Interaction with the 3D model

Rotate: Hold left mouse-button and move mouse.
Zoom: Hold right mouse-button and move mouse up or down or scroll.
Translate: Hold left and right mouse-buttons and move mouse.

Selection of preset views

Click on a view button to display the preset view as shown on the button.

Full screen mode

Enter full screen mode: Ctrl + L
Exit full screen mode: Esc

Immunohistochemistry and clinical data patient

Click on the tab “Clinical data patient” to display the data of the patient.

Technical notes

This PDF file should be viewed in Adobe Acrobat Reader X or higher. 3D interaction is only possible on MS Windows or Mac OS. Javascript and playing of 3D content must be enabled.
Open Edit, Preferences to ensure the following:

1) In JavaScript: enable Enable Acrobat JavaScript
2) In 3D & Multimedia: enable Enable playing of 3D content
3) In 3D & Multimedia, 3D Tool Options: disable Show 3D Orientation Axis
4) In 3D & Multimedia, Auto-Degrade Options, Optimization Scheme for Low Framerate: select None

Granuloma 5: Abscess granuloma (Rich focus)

- Granuloma
- Necrosis
- Giant cells
- Blood vessels
- Cerebellum
- Meninges

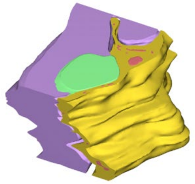
Overview

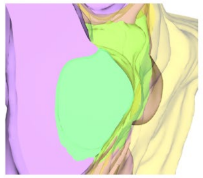
Rich focus

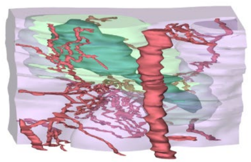
Blood vessels

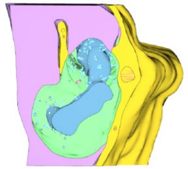
Granuloma layers

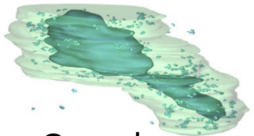
Granuloma

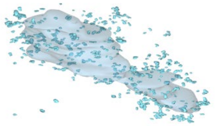
Giant cells

Amsterdam UMC
University Medical Centers

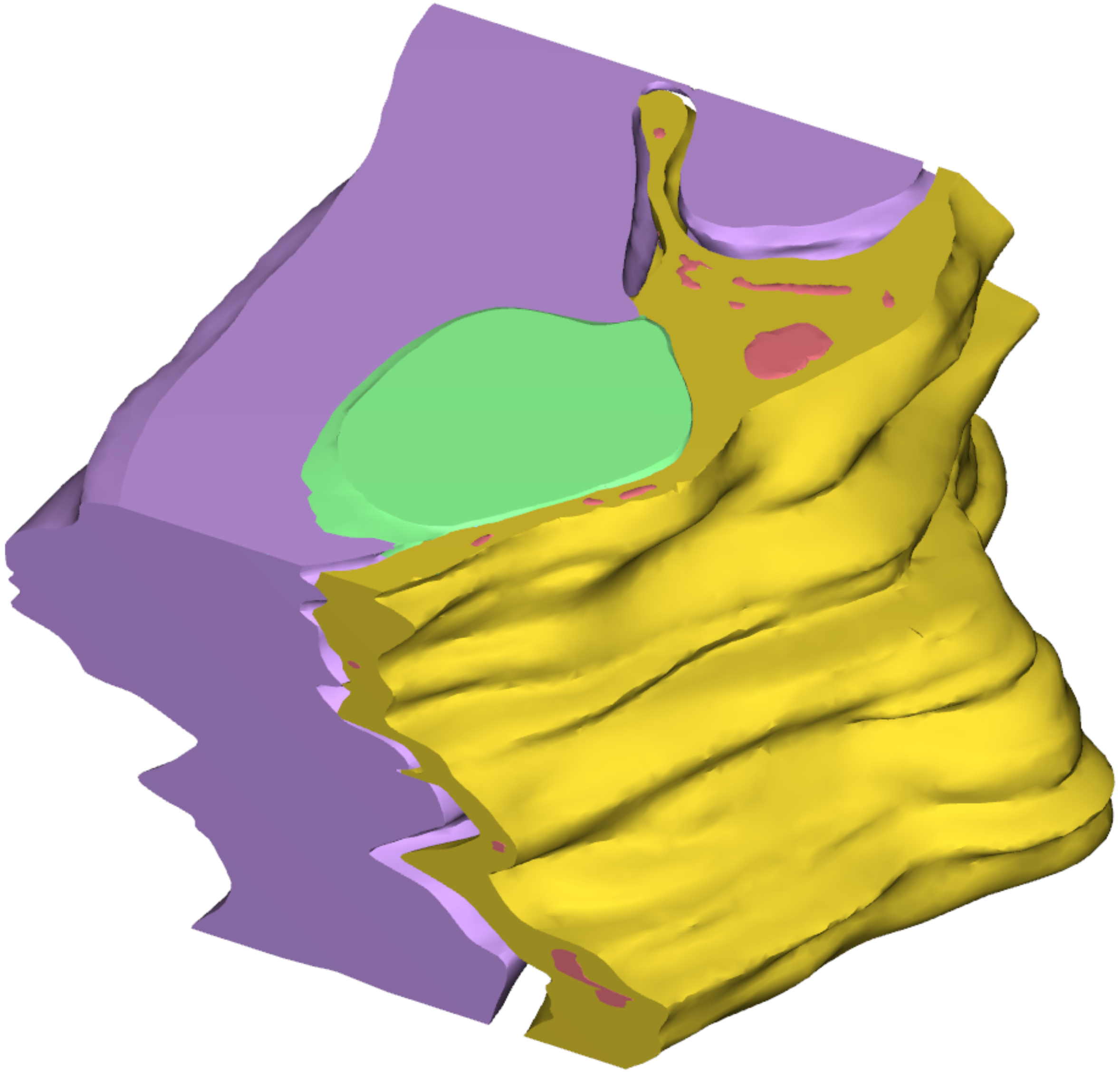

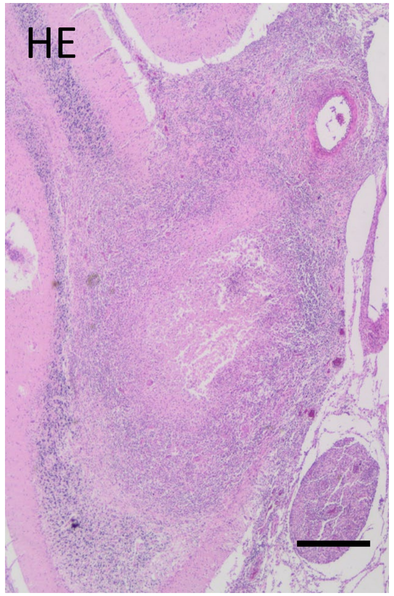

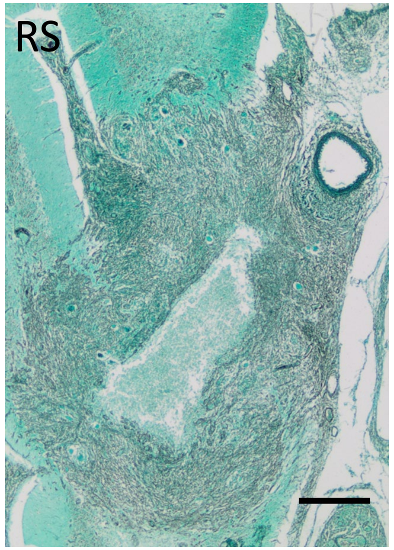

Scale bare = 200 µm

Clinical data of patient (case number 38)

Help | 3D model | Clinical data patient

**Background**	
Year of admission at hospital	1998
Age (month)	19 month
Sex	Female
**Clinical information**	
Initial presentation	Since 2 weeks fever, malaise, vomiting, weakness and seizures
Physical examination[b]	Glasgow Coma Scale 3/15, spastic quadriplegia, poorly reacting pupils
**Diagnosis**	Proven
TBM Stage[a]	Stage III
Lumbar puncture	Polymoph 18, lymphocytes 41, protein 4,0g/L, glucose 1.3 mmol/L, Ziehl-Neelsen: positive
Cerebral imaging	CT: bilateral caudate infarcts, enhancement basal cistern, periventricular low densities, hydrocephalus<br>Air encephalography: communicating hydrocephalus
**Treatment**	
Tuberculostatics	Rifampicin, Isoniazide, Pyrazinamide, Ethionamide
Other medication	Prednison, Acetazolamide, Furosomide, Thalidomide, Mebendazole, Diazepam
Duration	8 days
**Outcome**	Death after development of pneumonia
**Post mortem**	
Central Nervous system	Exudate present covering the surface of the brain particularly involving the basal cisterns. Horizontal sections of the brain showed widening of the ventricles. Histological sections confirm the presence of a granulomatous inflammatory process involving the leptomeninges. Exudative endarteritits was present with associated areas of infarction in the cerebral hemispheres, basal ganglia and brain stem. No organisms could be demonstrated by Ziehl-Nielson staining on histology.

a. Tuberculous meningitis stage is based on the ‘refined’ British Medical Research Council scale (van Toorn 2012)
b. Glascow coma scale reference
