# Supplementary material for: Three-dimensional visualizations from a dataset of immunohistochemical stained serial sections of human brain tissue containing tuberculosis related granulomas
Source: Data Brief. 2020 Nov 14;33:106532. doi: 10.1016/j.dib.2020.106532 (PMC7701168; doi:10.1016/j.dib.2020.106532)
Supplement: Supplementary file 1 [file mmc1.zip › Meningitis.pdf]

Information on the use of this interactive 3D-PDF

Meninges

Blood vessels

Cerebellum

Brainstem

Border zone inflammation

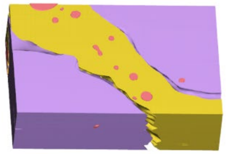

Overview

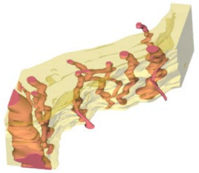

Blood vessels

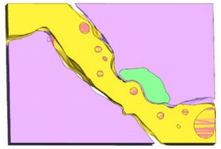

Border inflammation section

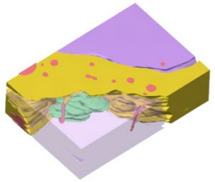

Border inflammation surface

Amsterdam UMC
University Medical Centers

Selection of structures

The top left panel contains buttons to show or hide structures, or to make them transparent.

button example:

show transparent hide

Interaction with the 3D model

Rotate: Hold left mouse-button and move mouse.
Zoom: Hold right mouse-button and move mouse up or down or scroll.
Translate: Hold left and right mouse-buttons and move mouse.

Selection of preset views

Click on a view button to display the preset view as shown on the button.

Full screen mode

Enter full screen mode: Ctrl + L
Exit full screen mode: Esc

Immunohistochemistry and clinical data patient

Click on the tab “Clinical data patient” to display the data of the patient.

Technical notes

This PDF file should be viewed in Adobe Acrobat Reader X or higher. 3D interaction is only possible on MS Windows or Mac OS. Javascript and playing of 3D content must be enabled.
Open Edit, Preferences to ensure the following:

1) In JavaScript: enable Enable Acrobat JavaScript
2) In 3D & Multimedia: enable Enable playing of 3D content
3) In 3D & Multimedia, 3D Tool Options: disable Show 3D Orientation Axis
4) In 3D & Multimedia, Auto-Degrade Options, Optimization Scheme for Low Framerate: select None

Meningitis with border inflammation of parenchyma

Help | 3D model | Clinical data patient

- Meninges
- Blood vessels
- Cerebellum
- Brainstem
- Border zone inflammation

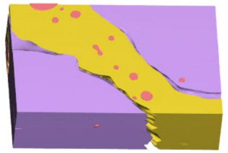
Overview

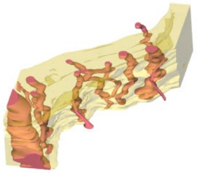
Blood vessels

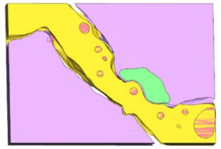
Border inflammation section

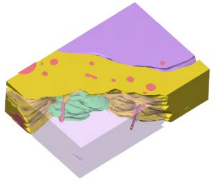
Border inflammation surface

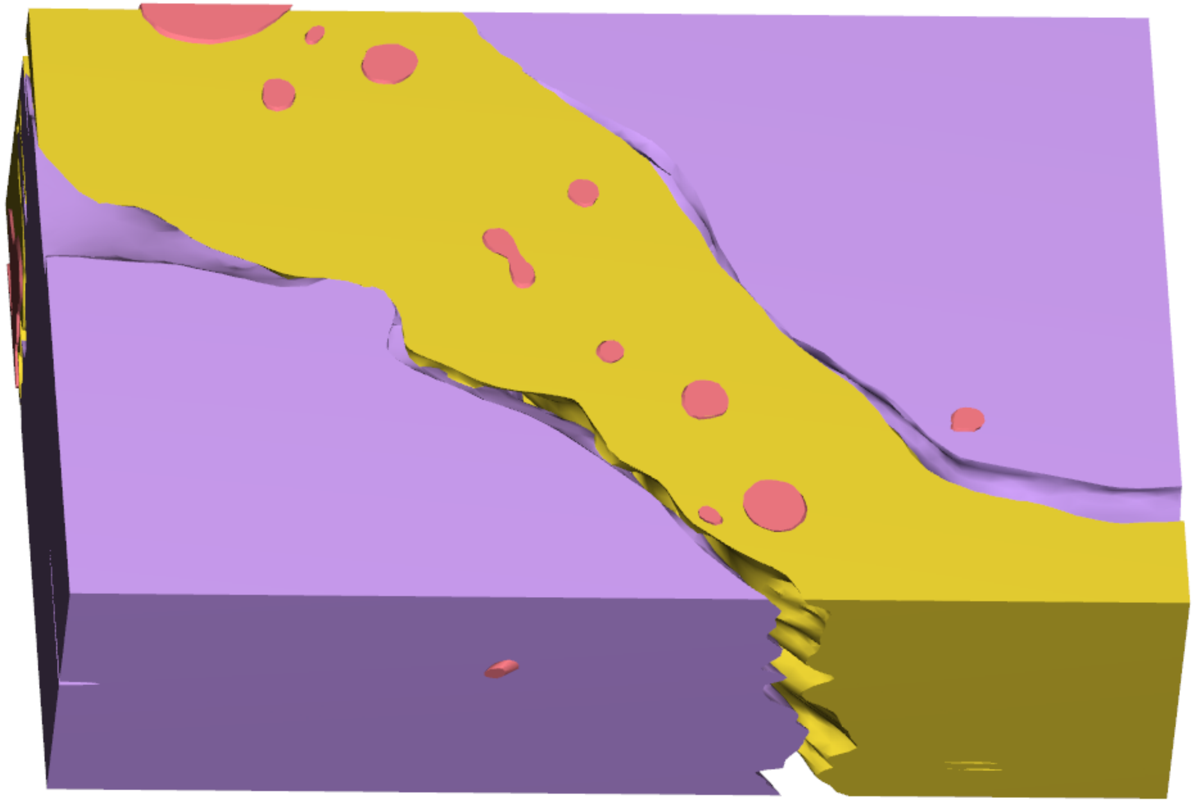

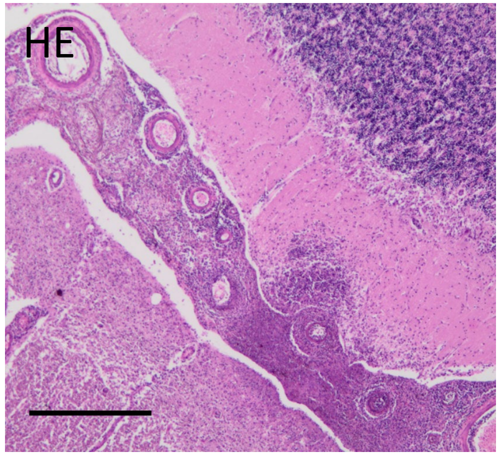

Scale bare = 200 µm

Clinical data of patient (case number 64)

Help | 3D model | Clinical data patient

Background	
Year of admission at hospital	1990
Age (month)	343 month (28 years)
Sex	Male
Clinical information	
Initial presentation	Frontal headache for 2 month, gradually deterioration of condition till unable to walk or speak at presentation
Physical examination	Depressed level of consciousness, generalised diminished reflexes, upgoing plantar reflexes, unresponsive pupils to light
Diagnosis	Probable
TBM Stage[a]	Stage III
Lumbar puncture	Polymph 27, lymphocytes 60, protein 2,2g/L , globulin 2+, glucose 0.7 mmol/L
Cerebral imaging	MRI of brain: hydrocephalus
Treatment	Therapy was ordered but patient suffered a cardiorespiratory arrest and died before therapy was started
Tuberculostatics	-
Other medication	-
Duration	0 days
Outcome	Death
Post mortem	
Central Nervous system	The brain showed congestion of leptomeningeal vessels with generalised swelling as indicated by gyral flattening. Small tubercles were scattered on the leptomeninges of the convexities, especially in the right parietal region. Examination of the base of the brain showed a possible early central herniation. A thick basal exudate was evident in the interpeduncular fossa extending up the Sylvian fissures bilaterally. The pons demonstrated an area of haemorrhagic infarction.
Other tissue	Lymphadenopathy with caseation was noted in the mediastinum as well as the peripancreatic nodes.

a. Tuberculous meningitis stage is based on the ‘refined’ British Medical Research Council scale (van Toorn 2012)
