# Supplementary material for: Three-dimensional visualizations from a dataset of immunohistochemical stained serial sections of human brain tissue containing tuberculosis related granulomas
Source: Data Brief. 2020 Nov 14;33:106532. doi: 10.1016/j.dib.2020.106532 (PMC7701168; doi:10.1016/j.dib.2020.106532)
Supplement: Supplementary file 1 [file mmc1.zip › Non-necrotizing granuloma.pdf]

Information on the use of this interactive 3D-PDF

Help | 3D model | Clinical data patient

Lymphocyte cuff
Giant cells
Blood vessels

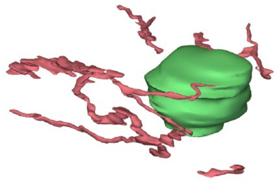

Overview

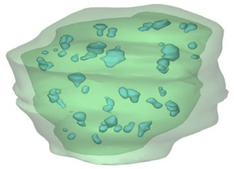

Granuloma

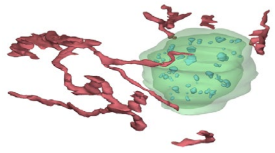

Blood vessels

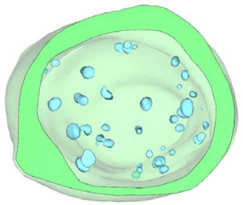

Granuloma layers

Selection of structures

The top left panel contains buttons to show or hide structures, or to make them transparent.

button example:

show transparent hide

Interaction with the 3D model

Rotate: Hold left mouse-button and move mouse.
Zoom: Hold right mouse-button and move mouse up or down or scroll.
Translate: Hold left and right mouse-buttons and move mouse.

Selection of preset views

Click on a view button to display the preset view as shown on the button.

Full screen mode

Enter full screen mode: Ctrl + L
Exit full screen mode: Esc

Immunohistochemistry and clinical data patient

Click on the tab "Clinical data patient" to display the data of the patient.

Technical notes

This PDF file should be viewed in Adobe Acrobat Reader X or higher. 3D interaction is only possible on MS Windows or Mac OS. Javascript and playing of 3D content must be enabled.
Open Edit, Preferences to ensure the following:

1) In JavaScript: enable Enable Acrobat JavaScript
2) In 3D & Multimedia: enable Enable playing of 3D content
3) In 3D & Multimedia, 3D Tool Options: disable Show 3D Orientation Axis
4) In 3D & Multimedia, Auto-Degrade Options, Optimization Scheme for Low Framerate: select None

Amsterdam UMC
University Medical Centers

Granuloma 2: Non-necrotizing granuloma

Help | 3D model | Clinical data patient

Lymphocyte cuff
Giant cells
Blood vessels

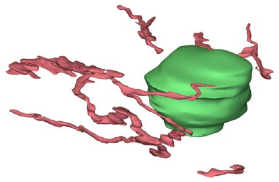
Overview

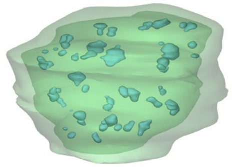
Granuloma

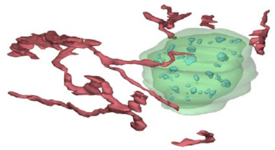
Blood vessels

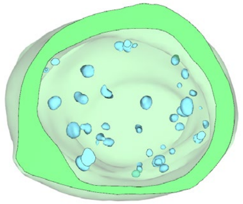
Granuloma layers

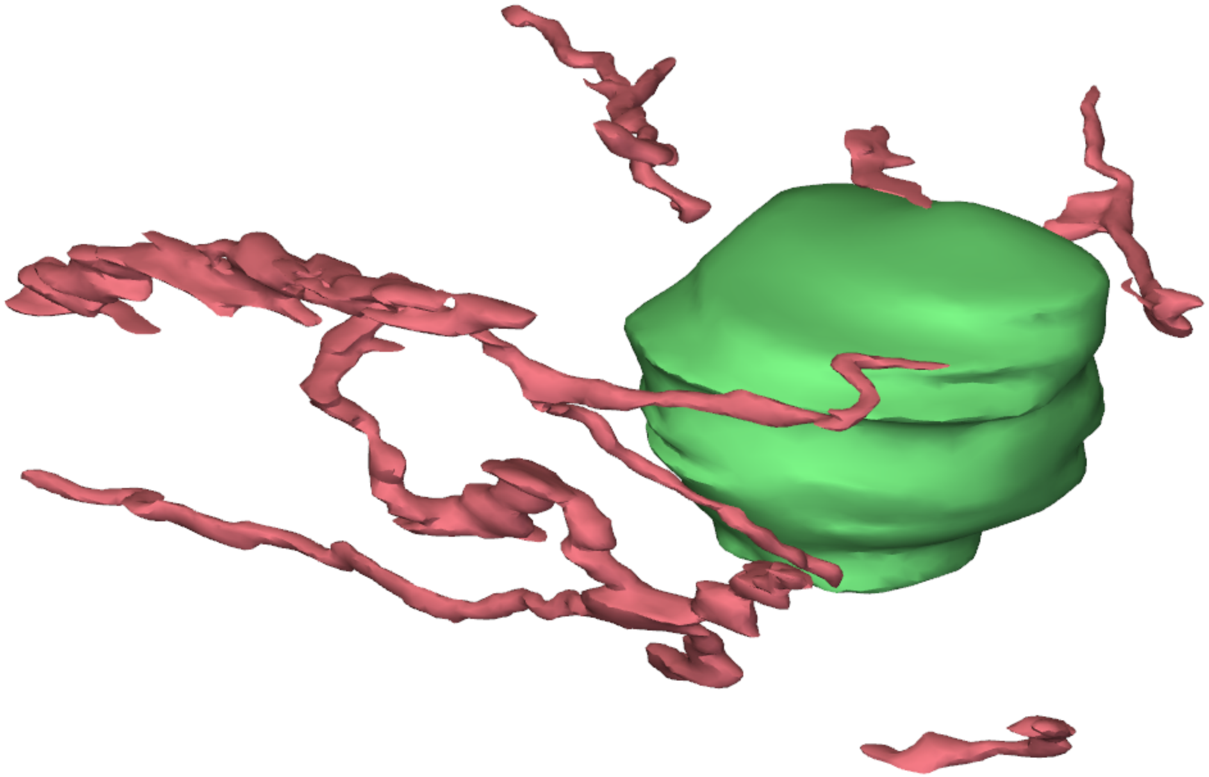

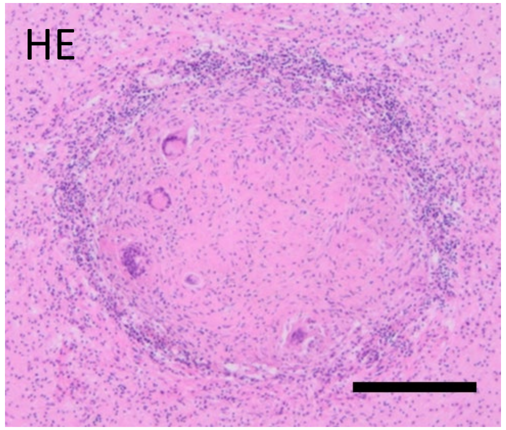

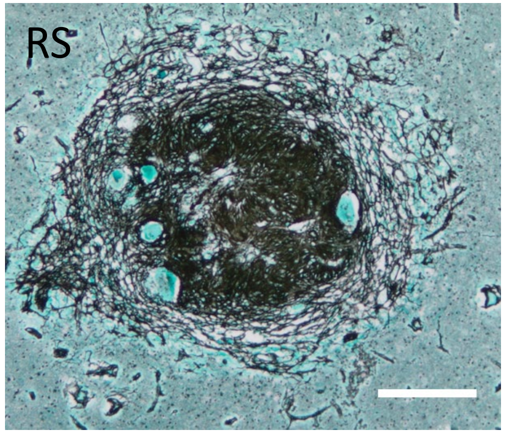

Scale bare = 100 µm

Clinical data of patient (case number 21)

Help | 3D model | Clinical data patient

Background	
Year of admission at hospital	1988
Age (month)	39 month
Sex	Female
Clinical information	
Initial presentation	Nausea since 2 weeks
Physical examination	Stuporose, decerebrated posture, generalised diminished reflexes, upgoing plantar reflexes at both side
Diagnosis	Proven
TBM Stage[a]	Stage III
Lumbar puncture	Polymorf 33, lymphocytes 44, protein 2,40 g/L , glucose 1.8 mmol/L, Ziehl-Neelsen: positive
Treatment	
Tuberculostatics	Rifampicine, Isoniazide, Pyrazinamide, Ethionamide, Penicilline G
Other medication	Furosemide, Acetazolamide, Chlooramfenicol
Duration	4 days
Outcome	Death
Post mortem	
Central Nervous system	Exudate present at the base of the brain. Tubercles were diffusely present over both hemispheres and were especially prominent fronto-temporal. Acid fast bacilli were present within the granulomas on histology.

a. Tuberculous meningitis stage is based on the ‘refined’ British Medical Research Council scale (van Toorn 2012)
